# The effects of vitamin E or lipoic acid supplementation on oxyphytosterols in subjects with elevated oxidative stress: a randomized trial

**DOI:** 10.1038/s41598-017-15615-y

**Published:** 2017-11-10

**Authors:** Sabine Baumgartner, Ronald P. Mensink, Guido R. Haenen, Aalt Bast, Christoph J. Binder, Otto Bekers, Constanze Husche, Dieter Lütjohann, Jogchum Plat

**Affiliations:** 1 Department of Human Biology, NUTRIM school of Nutrition and Translational Research in Metabolism, Maastricht University, Maastricht, The Netherlands; 2 Department of Toxicology, NUTRIM school of Nutrition and Translational Research in Metabolism, Maastricht University, Maastricht, The Netherlands; 3 Department of Laboratory Medicine, Medical University of Vienna, Vienna, Austria; 4 Department of Clinical Chemistry, Maastricht University Medical Centre+, Maastricht, The Netherlands; 5 Institute of Clinical Chemistry and Clinical Pharmacology, University of Bonn, Bonn, Germany

## Abstract

Despite increased serum plant sterol concentrations after consumption of plant sterol enriched margarines, plasma oxyphytosterol concentrations were not increased in healthy subjects. Here, we assessed plasma oxyphytosterol concentrations and whether they are affected by antioxidants in subjects with elevated oxidative stress. Twenty subjects with impaired glucose tolerance (IGT) or type 2 diabetes (DM2) consumed for 4 weeks placebo, vitamin E (804 mg/d) or lipoic acid capsules (600 mg/d). Plasma and blood cell oxyphytosterol and oxycholesterol concentrations were determined in butylated hydroxytoluene-enriched EDTA plasma via GC-MS. Also, markers reflecting oxidative stress and antioxidant capacity were measured. Plasma oxycampesterol and oxysitosterol concentrations were 122% and 83% higher in IGT or DM2 subjects than in healthy subjects, as determined in an earlier study. Vitamin E or lipoic acid supplementation did not reduce plasma oxyphytosterol and oxycholesterol concentrations, or other markers reflecting oxidative stress or antioxidative capacity. Concentrations of different oxyphytosterols correlated within plasma, and within red blood cells and platelets. However, plasma and blood cell oxyphytosterol levels did not correlate. Although plasma oxyphytosterol concentrations are higher in IGT or DM2 subjects than in healthy subjects, 4-weeks vitamin E or lipoic acid supplementation does not lower plasma oxycholesterol or oxyphytosterol concentrations.

## Introduction

Consumption of plant sterol enriched products lowers serum low-density lipoprotein cholesterol (LDL-C) concentrations, while at the same time serum plant sterol concentrations are increased. A recent meta-analysis showed that at recommended intakes of 1.6 g/d, serum sitosterol concentrations increase by 31% (CI: 26–37%) and campesterol concentrations by 37% (CI: 29–45)^1^. Whether these increased plant sterol concentrations have an impact on CVD risk has been subject of debate for many years now, as epidemiological studies have reported contradictive findings^2,3^. Previously, we postulated that this controversy might relate to the fact whether plant sterols are oxidized or not^4^. Cholesterol and plant sterols share structural similarities and can be oxidized to form either oxycholesterols (cholesterol oxidation products) or oxyphytosterols (plant sterol oxidation products)^5^. While oxycholesterol concentrations have been characterised as an oxidative stress marker^6^, information regarding oxyphytosterol metabolism and their effects on health in humans is scarce. In fact, there is a clear lack of knowledge regarding absorption, endogenous formation via (ROS-mediated) oxidation of circulating plant sterols, and potential effects of other dietary components on circulating serum oxyphytosterol concentrations. We have previously shown that increased dietary plant sterol consumption do not change fasting oxyphytosterol concentrations, despite elevated fasting plant sterol concentrations. However, postprandial oxyphytosterol concentrations increased after consumption of a high-fat meal enriched with plant sterol esters^7^. Information of dietary antioxidant supplementation on circulating oxyphytosterol concentrations in metabolically stressed subjects would expand our knowledge on oxyphytosterol metabolism in humans. Therefore, the objective of this study was to examine changes in plasma oxyphytosterol concentrations after 4 weeks vitamin E or lipoic acid supplementation in subjects with impaired glucose tolerance (IGT) or diabetes type 2 (DM2), who are characterized by increased oxidative stress^8,9^. These two antioxidants were chosen, since vitamin E is a typical fat-soluble antioxidant, while lipoic acid is fat- and water-soluble antioxidant and both have been shown to lower oxidative markers in type 2 diabetics^10,11^.

## Results

**Figure 1:**
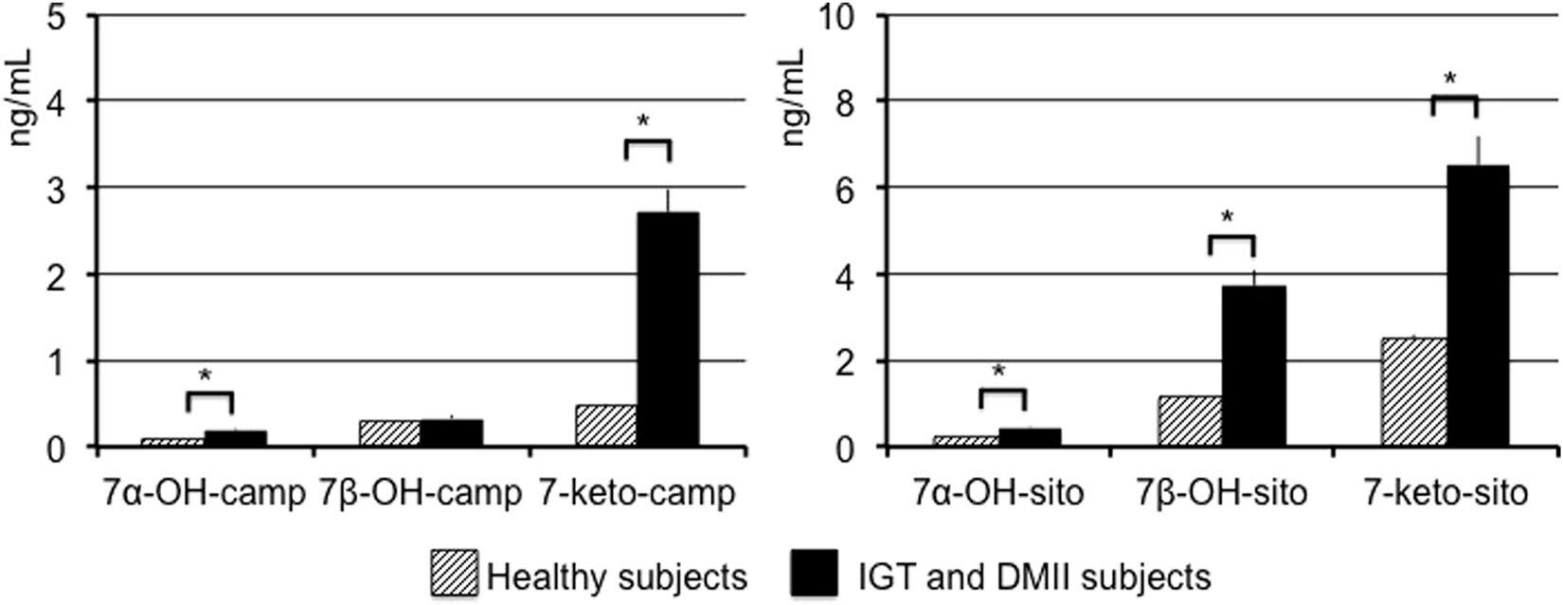
Fasting plasma oxyphytosterol concentrations after a control period in healthy subjects (n = 43) and subjects with IGT or type 2 diabetes (n = 20). Data are presented as mean ± SE, significantly different oxyphytosterol concentrations between the two groups *(P < 0.001). Data from healthy subjects are derived from Baumgartner *et al*.^4^ and data from subjects with IGT or type 2 diabetes are derived from the current study.

Average daily intakes of energy, fiber, and cholesterol, and the proportions of energy from protein, carbohydrates, total fat, saturated fatty acids, monounsaturated fatty acids, and polyunsaturated fatty acids, and habitual vitamin E intake in the background diet did not differ between the three periods (Supplemental Table 1). Plasma vitamin E concentrations were measured at the end of the three periods (Supplemental Table 2). Plasma vitamin E concentrations were 21.0 ± 9.2 μg/mL higher at the end of the vitamin E period (78%, P < 0.001) as compared with the control period and 20.3 ± 9.01 (72%, P < 0.001) higher as compared with the lipoic acid period. Lipoic acid has a short plasma half-life of approximately 30 minutes^12^ and since the last dose of lipoic acid capsules was taken at least 12 hours before blood sampling, lipoic acid concentrations were not determined in plasma. Fasting oxyphytosterol concentrations in subjects with IGT or type 2 diabetes at the end of the control period were compared with those from a previous study with healthy subjects during the control period^4^. Oxyphytosterol concentrations were comparable between subjects with IGT and type 2 diabetes. Figure 1 shows that 7α-OH-campesterol and 7α-OH-sitosterol were significantly higher in subjects with IGT or type 2 diabetes compared with healthy subjects (P < 0.001). 7β-OH-campesterol concentrations were comparable, while 7β-OH-sitosterol concentrations were higher in subjects with IGT or type 2 diabetes compared with healthy subjects (P < 0.001). Concentrations of 7-keto-campesterol and 7-keto-sitosterol were also significantly higher in subjects with IGT or type 2 diabetes compared with healthy subjects (P < 0.001). Indeed, these findings suggest that IGT and diabetic patients had increased oxidative stress. Fasting oxyphytosterol and oxycholesterol levels in plasma, RBC’s, and platelets in subjects with IGT or type 2 diabetes are shown in Table 1. In PBMC’s, oxyphytosterol levels were below the detection limit. Against our expectations, plasma 7α-OH-, 7β-OH- and 7-keto-cholesterol, -campesterol and -sitosterol concentrations were comparable at the end of the vitamin E, lipoic acid and control periods. Cholesterol-standardized oxyphytosterol and oxycholesterol levels showed similar results. In RBC’s, oxycholesterol concentrations were comparable during the vitamin E, lipoic acid and control periods. For the oxyphytosterols, 7-keto-campesterol median levels were lower in the vitamin E period as compared to the control period (37 (23–87) pg/mg; P = 0.014, while the other oxyphytosterol isoforms were comparable at the end of the three periods. In the PBMC fraction, 7α-OH-, 7β-OH- and 7-keto-cholesterol, -campesterol and -sitosterol levels were comparable in the vitamin E, lipoic acid and control periods. In platelets, 7β-OH-cholesterol median levels were higher in the vitamin E period compared to the control period (115.0 (33.9–536.4; P = 0.037) while the other cholesterol oxidation levels were comparable in the vitamin E, lipoic acid and control periods.

**Table 1:** Effect of vitamin E and lipoic acid supplementation on oxyphytosterol and oxycholesterol concentrations in plasma, RBC’s and platelets.

	Control period	Vitamin E period	Lipoic acid period
Plasma (ng/mL)			
7α-OH-cholesterol	48.83 ± 32.10	50.33 ± 31.13	52.97 ± 33.08
7α-OH-campesterol	0.20 ± 0.09	0.22 ± 0.12	0.18 ± 0.10
7α-OH-sitosterol	0.46 ± 0.23	0.51 ± 0.27	0.53 ± 0.21
7β-OH-cholesterol	8.82 ± 4.03	8.95 ± 4.07	8.32 ± 4.03
7β-OH-campesterol	0.33 ± 0.17	0.35 ± 0.27	0.35 ± 0.26
7β-OH-sitosterol	3.73 ± 1.63	4.05 ± 1.61	4.51 ± 2.21
7-keto-cholesterol	23.53 ± 10.36	26.44 ± 12.90	21.74 ± 7.76
7-keto-campesterol	2.71 ± 1.20	2.59 ± 1.15	2.81 ± 1.39
7-keto-sitosterol	6.50 ± 3.07	7.21 ± 3.82	7.70 ± 3.23
Sum oxycholesterol	80.64 ± 37.90	88.93 ± 40.54	83.03 ± 39.0
Sum oxyphytosterol	13.28 ± 5.22	14.93 ± 5.65	16.32 ± 6.61
RBC’s (oxyphytosterols: pg/mg, oxycholesterol: ng/mg)			
7α-OH-cholesterol	1.0 (0.3–24.8)	0.4 (0.2–1.0)	0.4 (0.2–19.4)
7α-OH-campesterol	2.5 (1.6–76.0)	2.3 (0.7–3.8)	6.2 (0.85–74.6)
7α-OH-sitosterol	8.8 (4.0–80.1)	7.2 (2.1–11.0)	6.8 (3.4–81.4)
7β-OH-cholesterol	1.4 (0.2–35.3)	0.4 (0.2–1.3)	0.4 (0.1–28.2)
7β-OH-campesterol	2.6 (0–76.8)	1.5 (0–3.6)	1.9 (0.1–109.0)
7β-OH-sitosterol	20.7 (11.5–122.7)	18.5 (4.5–24.8)	18.4 (3.7–127.0)
7-keto-cholesterol	3.3 (0.8–95.9)	1.3 (0.7–3.9)	1.1 (0.8–115.7)
7-keto-campesterol	51.0 (31.4–330.7)	36.7 (23.4-86.6)^1^	40.2 (23.7–301.7)
7-keto-sitosterol	91.3 (65.8–333.0)	89.0 (45.5–157.1)	81.7 (43.1–282.6)
Sum oxycholesterol	5.6 (1.6–156.0)	2.1 (1.1–6.1)	2.0 (1.3–161.4)
Sum oxyphytosterol	180.3 (24.9–992.2)	156.4 (76.4–271.3)	153.0 (75.3–885.1)
Platelets (pg/mg)			
7α-OH-cholesterol	72.6 (23.1–229.0)	86.2 (31.9–504.7)	107.6 (7.0–400.1)
7α-OH-campesterol	3.6 (0.9–18.2)	5.0 (1.8–26.2)	2.6 (1.1–13.8)
7α-OH-sitosterol	16.2 (5.2–63.4)	19.0 (5.2–98.4)	7.0 (5.1–65.3)
7β-OH-cholesterol	81.3 (24.6–328.5)	115.0 (33.9–536.4)^1^	104.0 (27.7–371.4)
7β-OH-campesterol	2.6 (1.2–15.0)	2.4 (0–23.2)	3.0 (0.5–15.1)
7β-OH-sitosterol	30.6 (4.1–101.0)	30.8 (4.6–145.8)	16.4 (2.8–122.5)
7-keto-cholesterol	345.4 (19.0–1068.6)	376.5 (36.9–1185.2)	234.1 (61.8–2110.9)
7-keto-campesterol	254.0 (71.2–757.0)	253.4 (63.5–1260.9)	112.0 (69.8–884.2)
7-keto-sitosterol	444.9 (152.8–1227.8)	469.8 (156.0–2075.6)	233.4 (155.2–1618.9)
Sum oxycholesterol	560.4 (66.7–1472.5)	642.0 (102.6–2104.7)	465.1 (112.7–2833.8)
Sum oxyphytosterol	752.5 (245.9–2169.4)	779.6 (234.8–3564.5)	357.5 (248.6–2670.8)
PBMC’s (pg/mg)			
7α-OH-cholesterol	292 (57–6840)	463 (65–5202)	227 (63–1895)
7β-OH-cholesterol	174 (22–16 997)	223 (2–9925)	178 (19–9288)
7-keto-cholesterol	184 (97–666)	183 (49–1014)	192 (65–81 762)
Sum oxycholesterol	999 (282–24 078)	1147 (2–15 471)	1117 (402–82 870)

Values are means ± SD or median (ranges) (n = 20). ^1^Significantly different compared with control period (P < 0.05). Exact P-values are described in the text.

Table 2 shows markers reflecting the level of oxidative stress and antioxidative capacity. OxPL-apoB levels and MDA concentrations, which are both markers of oxidative stress, were comparable at the end of the three periods. In addition, parameters reflecting antioxidative capacity did not differ between the vitamin E, lipoic acid and control periods. Also plasma cytokines, cellular adhesion molecules, and iron and copper status were comparable between the three periods **(**Supplemental Table 3 **)**. Plasma glucose, HbA1c, FGF-19, FGF-21, serum plant sterols and lathosterol and cholestanol concentrations were comparable between the vitamin E, lipoic acid and control periods (Table 3). Serum total cholesterol concentrations were 0.26 ± 0.35 mmol/L higher at the end of the vitamin E period (4.9%; P = 0.021) as compared with the lipoic acid period and 0.23 ± 0.44 mmol/l (4.2%; P = 0.021) higher as compared with the control period. This was also reflected in the total cholesterol/HDL cholesterol ratio, which was increased in the vitamin E period by 0.27 ± 0.40 (5.6%; P = 0.007) compared with the control period, while there was a trend towards reduction in the lipoic acid period (−4.4%; P = 0.062). Also serum apoB100 concentrations were higher in the vitamin E period compared with the lipoic acid (0.05 ± 0.08 g/L; P = 0.048) and control periods (0.07 ± 0.10 g/L P = 0.001). Finally, apoA1 concentrations were also increased in the vitamin E period compared with lipoic acid (0.06 ± 0.08 g/L; P = 0.004) and control periods (0.05 ± 0.09 g/L; P = 0.025). No differences were found in serum TAG and HDL-C concentrations between the three periods.

**Table 2:** Effect of vitamin E and lipoic acid supplementation on oxidative and antioxidative parameters.

	Control period	Vitamin E period	Lipoic acid period
OxLDL (oxidized phospholipids:apoB100 ratio)	0.05 ± 0.07	0.04 ± 0.06	0.04 ± 0.06
MDA (μmol/L)	2.2 ± 0.4	2.1 ± 0.4	2.0 ± 0.3
Trolox equivalent (μmol/L)	489.5 ± 73.7	479.3 ± 77.8	493.6 ± 90.1
GSH/GSSG	49.4 ± 30.8	44.7 ± 22.8	50.0 ± 27.2
Uric acid (μmol/L)	312.4 ± 59.8	313.5 ± 59.4	321.3 ± 70.4

Values are means ± SD (n = 20).

**Table 3:** Effect of vitamin E and lipoic acid supplementation on plasma glucose, serum lipid and lipoprotein concentrations and serum plant sterol, lathosterol and cholestanol concentrations.

	Control period	Vitamin E period	Lipoic acid period
Glucose (mmol/L)	7.26 ± 1.64	7.29 ± 1.68	7.16 ± 1.47
HbA1c (%)	6.18 ± 0.86	6.19 ± 0.84	6.22 ± 0.85
FGF-19 (pg/mL)	46 ± 45	49 ± 47	50 ± 54
FGF-21 (pg/mL)	400 ± 263	486 ± 278	437 ± 227
Total cholesterol (mmol/L)	5.28 ± 0.97	5.52 ± 0.90^1,3^	5.28 ± 0.96
LDL cholesterol (mmol/L)	3.30 ± 0.89	3.44 ± 0.90^3^	3.21 ± 0.86
HDL cholesterol (mmol/L)	1.34 ± 0.27	1.30 ± 0.25	1.30 ± 0.25
Total cholesterol/HDL	4.12 ± 0.90	4.38 ± 1.01^2^	4.18 ± 0.89
Triacylglycerol (mmol/L)	1.62 ± 0.64	1.72 ± 0.65	1.73 ± 0.66
ApoB100 (g/L)	1.04 ± 0.19	1.11 ± 0.20^2,3^	1.06 ± 0.19
ApoA1 (g/L)	1.41 ± 0.22	1.45 ± 0.20^1,4^	1.40 ± 0.22
Sitosterol*	1.40 ± 0.83	1.48 ± 0.84	1.40 ± 0.72
Campesterol*	2.03 ± 1.11	2.08 ± 1.17	2.00 ± 1.12
Lathosterol*	1.64 ± 0.62	1.60 ± 0.49	1.54 ± 0.53
Cholestanol*	2.07 ± 0.37	2.05 ± 0.35	2.04 ± 0.34

Values are means ± SD and *sterols are expressed as μmol/mmol cholesterol (n = 20). Significantly different compared with control period: ^1^(P < 0.05), ^2^(P < 0.01). Significantly different compared with lipoic acid period: ^3^(P < 0.05), ^4^(P < 0.01). Exact P-values are described in the text.

### Correlations

In the control period, positive correlations were found between plasma 7α-OH-campesterol and 7α-OH-sitosterol concentrations (r = 0.845; P < 0.001), between 7β-OH-campesterol and 7β-OH-sitosterol concentrations (r = 0.625; P = 0.004), and between 7-keto-campesterol and 7-keto-sitosterol concentrations (r = 0.902; P < 0.001). Although plasma concentrations between the different oxyphytosterol isoforms also correlated significantly, correlations were less strong than between similar isoforms. Also in RBC’s and platelets, oxycampesterol and oxysitosterol levels were correlated, although stronger correlations were seen in platelets compared with RBC’s (Table 4). Plasma and RBC oxyphytosterol levels, plasma and platelet oxyphytosterol levels and RBC and platelets oxyphytosterol levels did not correlate.

**Table 4:** Oxyphytosterol correlations within plasma and within tissue (RBC’s & platelets) in control period (n = 20).

Parameter	Parameter	Compartment	Correlation*	P-value
7α-OH-campesterol	7α-OH-sitosterol	Plasma	0.845	<0.001
7β-OH-campesterol	7β-OH-sitosterol	Plasma	0.625	<0.01
7keto-campesterol	7keto-sitosterol	Plasma	0.902	<0.001
7α-OH-campesterol	7β-OH-campesterol	Plasma	0.442	0.058
	7keto-campesterol	Plasma	−0.408	0.083
7β-OH-campesterol	7keto-campesterol	Plasma	−0.071	0.773
7α-OH-sitosterol	7β-OH-sitosterol	Plasma	0.678	<0.01
	7keto-sitosterol	Plasma	−0.067	0.785
7β-OH-sitosterol	7keto-sitosterol	Plasma	0.538	<0.015
7α-OH-campesterol	7β-OH-campesterol	RBC	0.922	<0.001
	7keto-campesterol	RBC	0.390	0.110
7β-OH-campesterol	7keto-campesterol	RBC	0.420	0.080
7α-OH-sitosterol	7β-OH-sitosterol	RBC	0.798	<0.001
	7keto-sitosterol	RBC	0.440	0.07
7b-OH-sitosterol	7keto-sitosterol	RBC	0.340	0.152
7α-OH-campesterol	7β-OH-campesterol	Platelets	0.577	<0.01
	7keto-campesterol	Platelets	0.746	<0.001
7β-OH-campesterol	7keto-campesterol	Platelets	0.653	<0.01
7α-OH-sitosterol	7β-OH-sitosterol	Platelets	0.809	<0.001
	7keto-sitosterol	Platelets	0.741	<0.001
7β-OH-sitosterol	7keto-sitosterol	Platelets	0.794	<0.001

*Correlations in plasma concentrations were determined with Pearson’s correlation and correlations in RBC and platelet levels were determined with Spearman’s rho correlation. Exact P-values are described in the text.

In plasma, 7α-OH-cholesterol correlated with 7α-OH-campesterol (r = 0.535; P = 0.018), but not with 7α-OH-sitosterol concentrations (r = 0.288; P = 0.232). In addition, 7β-OH-cholesterol correlated with 7β-OH-campesterol (r = 0.502; P = 0.029) and tended to correlate with 7β-OH-sitosterol concentrations (r = 0.407; P = 0.084). There were no correlations between 7-keto-cholesterol and 7-keto-plant sterol concentrations and OxPl-apoB levels only correlated with 7-keto-cholesterol concentrations.

## Discussion

There is a clear lack of knowledge on factors affecting oxyphytosterol concentrations in plasma and tissues. Earlier we have shown that consumption of plant sterol enriched products did not increase fasting plasma oxyphytosterol concentrations, despite increases in fasting serum non-oxidized plant sterol concentrations^4^. Here we demonstrate that circulating oxyphytosterol concentrations are significantly higher in subjects with IGT or type 2 diabetes as compared to healthy controls. The current population of IGT and diabetic subjects can therefore be classified as ‘oxidative stressed’, since they also had higher plasma oxycholesterol concentrations as healthy subjects^13^, elevated plasma MDA concentrations^14^ and lower TEAC values in comparison to healthy subjects^15^. This study also shows that these higher circulating oxyphytosterol concentrations were not reduced after four weeks consumption of vitamin E or lipoic acid. In addition, none of the other oxidative stress markers or parameters reflecting anti-oxidative capacity were changed after consuming these antioxidants, which was unexpected. Lipoic acid has a short plasma half-life of about 30 minutes and because the last dose of lipoic acid was taken at least 12 hours before blood sampling, lipoic acid concentrations were not determined in plasma. Nevertheless, in view of the crossover design, it can be assumed that all subjects adhered to the interventions as estimated by pill count and increased vitamin E concentrations after vitamin E intake. Therefore, the remaining question is why these two antioxidants did not have an effect. Potential factors that could have affected the outcome are population, dose and duration of the intervention period. Both vitamin E and lipoic acid have been shown to reduce oxidative stress markers in various populations in both short-term (<6 weeks) and in longer-term studies^10,16^. For example, vitamin E consumption (400 mg/d) for 3 months reduced MDA concentrations in a study with 80 type 2 diabetic patients^17^. In patients with carotid atherosclerosis 6 weeks of vitamin E (900 mg/d) supplementation reduced 7β-OH-cholesterol levels^18^. Plasma MDA and oxycholesterol concentrations were also measured in our study, but were not changed after high-dose vitamin E supplementation. As reviewed by Pazdro *et al*., most studies have demonstrated that vitamin E protects against lipid peroxidation in diabetic subjects, while effects on protein and DNA oxidation are less clear^19^. Intravenous lipoic acid therapy (600 mg/d) has been shown to reduce MDA and prostaglandin concentrations already after two weeks in 13 obese subjects with impaired glucose tolerance^20^, while 8 weeks oral lipoic acid supplementation (1200 mg/d) in obese subjects failed to reduce oxLDL and prostaglandin concentrations^21^. This may suggest that intravenous lipoic acid supplementation is more effective than oral supplementation in human subjects^21^, which might explain the lack of effect in our study. However, Marangon *et al*. used a comparable oral dose of lipoic acid, e.g. 600 mg/d and demonstrated a decrease in urinary isoprostane concentrations and a reduction in LDL oxidative susceptibility in 31 healthy subjects^11^. Their intervention period lasted 2 months and it might be postulated that our intervention period of 4 weeks was too short to induce changes in oxidative stress markers with an oral supplementation protocol. This overview illustrates that there is no clear explanation for the lack of effect when considering population, dose and study duration. Besides evaluating effects on oxidative parameters we also measured a panel of markers reflecting antioxidative capacity. Previous studies have shown an increase in endogenous antioxidant enzyme activities after vitamin E consumption (400 mg/d) in type 2 diabetic subjects^17,22^. In addition, lipoic acid increased intracellular glutathione concentrations in a variety of cell types and tissues^23,24^. However, we did not demonstrate an improvement in these plasma anti-oxidative markers after vitamin E or lipoic acid consumption. The present study was performed in a population with IGT or type 2 diabetes, subjects that are generally recognized for their increased oxidative stress^25^. Compared to the healthy population (n = 43), the IGT and diabetic subjects (n = 20) were older (63 ± 6 vs. 41 ± 18 years) and had a higher BMI (29.5 ± 3.7 vs. 24.8 ± 2.8 kg/m^2^) and it might be possible that these differences have also contributed to the elevated oxyphytosterol concentration in the current (pre-) diabetic population. However, oxidative stress plays an important role in the initiation and progression of DM2, and type 2 diabetic patients have increased plasma oxidized LDL-C and oxycholesterol concentrations^9^. We here demonstrate for the first time that indeed not only oxycholesterol, but also plasma oxyphytosterol concentrations appear to be higher in subjects with IGT or type 2 diabetes as compared with healthy subjects. Circulating oxycampesterol concentrations were 122% higher and oxysitosterol concentrations were 83% higher in IGT and type 2 diabetics as compared to control subjects, while serum plant sterol concentrations were comparable between the two populations. Except for 7β-OH-campesterol concentrations, almost all oxyphytosterol isoforms were higher in IGT and diabetic subjects.

Whether increased oxyphytosterol concentrations are potentially atherogenic was not the aim of the study, and remains to be determined. If so, it would be of interest to further characterize oxyphytosterol concentrations in different (diseased) populations and assess which dietary interventions might be successful in reducing plant sterol oxidation products. An important question is not only whether plasma oxyphytosterol concentrations are changed after vitamin E or lipoic acid, but also what happens with oxyphytosterol concentrations inside tissues. Luister *et al*. determined oxyphytosterol concentrations in plasma and aortic valve cusps in patients undergoing elective aortic valve replacement. They showed that patients with coronary artery disease (CAD) had higher plasma cholesterol-standardized oxysitosterol concentrations and higher oxycampesterol concentrations in aortic valve cusps^26^. The authors did not compare plasma and tissue oxyphytosterol concentrations between subjects with and without diabetes but these findings do indicate that plasma and tissue oxyphytosterol concentrations might also be increased in subjects with CAD. In addition, Schött *et al*. showed that in this population, oxyphytosterol isoforms correlated significantly within plasma and within tissue, while a correlation between plasma and aortic tissue was lacking^27^. These results are (partly) in agreement with our study, since we also observed no relation between plasma and blood cell oxyphytosterol concentrations, while the different oxyphytosterol isoforms within the various cells types did correlate. A potential explanation for the lack of correlation between plasma and tissue levels might be that plant sterols are oxidized within tissues after uptake and do not originate from uptake via plasma. Nevertheless, vitamin E and lipoic acid also failed to reduce oxyphytosterol and oxycholesterol values in RBC’s and platelet fractions, suggesting that antioxidants also do not affect oxyphytosterol concentrations in tissues.

Rather unexpected findings in our study were increased serum lipid and (apo)lipoprotein concentrations during the vitamin E period compared with lipoic acid and control periods. This effect was consistent, since both cholesterol (total and LDL-C) and its carrier (apoB100) concentrations were increased. ApoA1 concentrations were increased in the vitamin E period, although serum HDL-C concentrations were comparable in all periods. This cholesterol-raising effect of vitamin E was unanticipated and has only been described a few times before^10,28^. Fats in the vitamin E capsules cannot explain the observed effect, since the capsules provided minor amounts of linoleic and oleic acid. Changes in dietary intake are also highly unlikely, since food frequency questionnaires showed comparable dietary intake in all three periods. For now, we have no obvious explanation for this unexpected effect of vitamin E supplementation.

We have demonstrated that oxyphytosterol concentrations are higher in IGT and type 2 diabetic subjects than in healthy subjects and that circulating oxyphytosterols are not reduced by vitamin E and lipoic acid supplementation in oxidative or metabolically stressed subjects. The possible clinical implications of higher oxyphytosterol concentrations in IGT or type 2 diabetic subjects remain to be determined.

## Methods

### Study population

The study population consisted of 20 men and women with IGT or type 2 diabetes. Subjects attended two screenings visits to assess their eligibility before entering the study. These criteria included an age between 18 and 75 years, BMI between 20 and 35 kg/m^2^, no active cardiovascular disease or severe medical conditions that might interfere with the study, no use of insulin or lipid-lowering medication, mean serum total cholesterol <8.0 mmol/L, mean serum triacylglycerol <3.0 mmol/L, diagnosed with diabetes mellitus type 2 on a clinical basis or IGT. The diagnosis of type 2 diabetes or IGT was based on the use of oral hypoglycemic agents or an oral glucose tolerance test. Only subjects without diagnosed diabetes underwent an oral glucose tolerance test to verify potential presence of impaired glucose tolerance. For this, subjects consumed 82.5 gram of dextrose monohydrate dissolved in 250 mL of tap water and plasma glucose concentrations were measured after 2 hours. A value between 7.8–11.1 mmol/L was defined as having IGT. Twenty subjects of which 8 subjects with type 2 diabetes and 12 subjects with IGT were enrolled and completed the study. Baseline characteristics are shown in Supplemental Table 4. All participants gave their written informed consent for the study. The protocol was approved by the medical ethical committee of the Maastricht University Medical Centre+ (MUMC+) and was carried out in accordance with the approved guidelines. The trial is registered at clinicaltrials.gov as NCT01984567 on November 8, 2013.

### Diets and design

The study had a randomized placebo-controlled cross-over design and consisted of three intervention periods of 4 weeks, separated by washout periods of 4 weeks. Subjects were randomly allocated to the intervention periods, based upon a computer-generated table with random numbers. During each period, subjects were asked to consume daily either three placebo capsules, three capsules containing RRR-vitamin E (268 mg three times; 804 mg/day) or three capsules containing R-lipoic acid (200 mg three times; 600 mg/day). Commercially available soft gel vitamin E capsules were provided as d-α tocopherol acetate, containing soybean oil with a capsule shell consisting of gelatin (De Tuinen, the Netherlands). Lipoic acid powder was provided by the Richardson Centre for Functional Foods and Nutraceuticals (Winnipeg, Canada) and encapsulated with hard gelatin capsules by Basic Pharma (Geleen, the Netherlands). Subjects were instructed to consume one capsule during each meal with a glass of water. They visited the university at the start of each period, and again after 3 and 4 weeks of intervention and were provided with at least the amount of capsules needed for 4 weeks and excess capsules were returned at the end of each intervention period. Subjects completed a validated food frequency questionnaire (FFQ) at the end of each intervention periods and a dietician checked these questionnaires to calculate energy and nutrient intakes over the previous 4 weeks using the Dutch food composition table. Subjects were asked not to change their habitual diet, level of physical exercise or use of alcohol throughout the entire study.

In a previous placebo-controlled intervention study^4^, we measured fasting oxyphytosterol concentrations in healthy subjects after consumption of plant sterol ester enriched margarine, plant stanol ester enriched margarine and a control margarine. Information on plasma oxyphytosterol concentrations in humans is very scarce and therefore, we compared oxyphytosterol concentrations in the current population of IGT and type 2 diabetic subjects with those from the previous study in healthy subjects during the control period. This cross-sectional comparison will provide more information on plasma oxyphytosterol concentration in different populations.

### Blood sampling

Blood was sampled after an overnight fast at the start of each intervention period and after 3 and 4 weeks of intervention. The same person performed all venipunctures at approximately the same time of the day at the same day of the week. A clotting tube (Becton, Dickinson and Company, Franklin Lakes, NY, USA) was sampled at each occasion to prepare serum by low-speed centrifugation at 1300 g for 15 min at room temperature, at least half an hour after venipuncture. Serum was stored at −80 °C and used for analysis of lipid and (apo)lipoprotein concentrations, hsCRP concentrations, iron status parameters (total iron, ferritin and transferrin), copper status parameters (i.e. copper and ceruloplasmin), inflammatory markers and cellular adhesion molecules. EDTA tubes (Becton, Dickinson and Company, Franklin Lakes, NY, USA) were sampled at the end of each period in weeks 3 and 4 to prepare plasma by low-speed centrifugation at 1300 g for 15 min at 4 °C. To avoid auto-oxidation, 10 ml butylated hydroxytoluene (BHT; 25 mg/mL ethanol) was added per 1 mL of EDTA plasma, immediately after centrifugation and then stored at −80 °C. BHT-enriched plasma was used for analysis of oxyphytosterol, oxycholesterol, oxidized LDL and malondialdehyde (MDA) concentrations. Fresh whole EDTA blood was sampled in week 4 and was used for the isolation of red blood cells (RBCs). BHT (25 mg/mL ethanol) was added to the RBC and PBMC fraction, stored at −80 °C and used for the analysis of oxyphytosterol and oxysterol concentrations. Heparin tubes (Becton, Dickinson and Company, Franklin Lakes, NY, USA) were also sampled in week 4 to prepare plasma by low-speed centrifugation at 1300 g for 15 min at 4 °C. Heparin plasma was used for the analyses of the trolox equivalent antioxidant capacity (TEAC) assay, uric acid and vitamin E concentrations. For this, 10% TCA was added to the plasma (1:1) followed by centrifugation (13.000 rpm, 5 min at 4 °C). Whole heparin blood was sampled in week 4 and 1.3% 5-Sulfosalicylic Acid (SSA) in 10 mM HCL was added for the analysis of GSH/GSSG. Fresh whole heparin blood was used for the analysis of HbA1c concentrations. An Acid Citrate Dextrose (ACD) tube was sampled at the end of each period and was used for the isolation of blood platelets. BHT was added to the platelets fraction, stored at −80 °C and used for the analysis of oxyphytosterol and oxycholesterol concentrations. Finally a NaF tube was sampled at the end of each period and to prepare plasma by low-speed centrifugation at 1300 g for 15 min at 4 °C. NaF plasma was stored at −80 °C and was used for the analysis of glucose concentrations.

### Analyses

Lipid, (apo)lipoprotein, hsCRP and glucose concentrations were determined as previously described (4). Glycated hemoglobin (HbA1c) was measured by high-pressure liquid chromatography (HPLC) (Bio-Rad variant 2, Bio-Rad Laboratories, Inc.). Sitosterol and campesterol concentrations were analyzed using gas-liquid chromatography-flame ion detection (GC-FID) as described previously^29^. Oxyphytosterol concentrations (7α-hydroxy(OH)-campesterol, 7α-OH-sitosterol, 7β-OH-campesterol, 7β-OH-sitosterol, 7-keto-campesterol and 7-keto-sitosterol) and oxycholesterol concentrations (7α-OH-cholesterol, 7β-OH-cholesterol and 7-keto-cholesterol) in plasma as well as in red blood cells (RBCs), peripheral blood mononuclear cells (PBMCs) and platelets were analyzed by gas-liquid chromatography-mass spectroscopy (GC-MS) according to the procedure as described by Husche *et al*.^30^. Oxidized phospholipids on apolipoprotein B100 particles (OxPL-apoB) was measured by chemiluminescent ELISA as measurement for oxidized LDL (oxLDL)^31^. Iron and copper status parameters, malondialdehyde (MDA) concentrations (oxidative stress marker), trolox equivalent antioxidant capacity, GSH/GSSG, uric acid concentrations (antioxidative markers) and vitamin E concentrations were analyzed as previously described^15,32,33^. Plasma inflammatory markers (Il-6 and TNFα), cellular adhesion molecules (E-selectin, P-selectin, soluble (s) ICAM-1, sICAM-3 and sVCAM-1), thrombomodulin and serum amyloid A (SAA) were measured with a commercially available multi spot ELISA kit (Meso Scale Discovery). Fibroblast Growth Factor (FGF)-21 and FGF-19 concentrations were measured with a single spot ELISA kit (R&D systems). All analyses were performed in samples from week 4, except for lipid, (apo)lipoprotein and hsCRP concentrations which were measured in weeks 3 and 4 and averaged for data analysis.

### Statistics

It was calculated that 18 subjects needed to be included in this study to have a power of 80% with a P < 0.017 (to account for multiple comparisons between groups) to detect a change of 0.15 ng/mL in plasma 7β-OH-campesterol concentrations with a within-subject variation of 0.14 ng/mL. Since the expected dropout rate was 10%, 20 men and women were included in the study. A Shapiro-Wilk test for normality was performed to assess whether parameters followed a normal distribution. Effects of vitamin E or lipoic acid supplementation were evaluated by a univariate analysis of variance (ANOVA) with subject number as a random factor for normally distributed data. Differences in oxyphytosterol and oxycholesterol levels in RBC, PBMC and blood platelet fractions were evaluated by Friedman’s test for not normally distributed data and reported as medians with ranges. Oxyphytosterol concentrations in healthy subjects were measured in our previous study using exactly the same method^4^ and compared to the current population of IGT and diabetic subjects by a Mann-Whitney *U* test. All statistical analyses were performed two-sided using SPSS 20.0 for Mac Os X and adjusted by Bonferroni’s correction for multiple comparisons or by post-hoc analysis for Friedman’s test when appropriate (SPSS Inc., Chicago, IL, USA).

## Electronic supplementary material

Supplementary info

CONSORT

PROTOCOL
