# Supplementary material for: The effects of vitamin E or lipoic acid supplementation on oxyphytosterols in subjects with elevated oxidative stress: a randomized trial
Source: Sci Rep. 2017 Nov 10;7:15288. doi: 10.1038/s41598-017-15615-y (PMC5681676; doi:10.1038/s41598-017-15615-y)
Supplement: Supplementary file 1 — Supplementary info [file 41598_2017_15615_MOESM1_ESM.doc]

**SUPPLEMENTARY INFORMATION**

**The effects of vitamin E or lipoic acid supplementation on oxyphytosterols in subjects with elevated oxidative stress: a randomized trial**

Sabine Baumgartner, Ronald P. Mensink, Guido R. Haenen, Aalt Bast, Christoph Binder, Otto Bekers, Constanze Husche, Dieter Lütjohann, Jogchum Plat

|  | Control period | Vitamin E period | Lipoic acid period |
| --- | --- | --- | --- |
| Energy (MJ) | 8.3 ± 2.8 | 8.3 ± 2.3 | 8.3 ± 2.3 |
| Fat (energy%)  SAFA^1^  MUFA^1^  PUFA^1^ | 38.2 ± 7.4  11.8 ± 3.1  13.6 ± 2.9  9.0 ± 3.8 | 37.0 ± 6.5  11.6 ± 2.3  13.4 ± 3.2  8.4 ± 2.6 | 36.7 ± 6.7  12.1 ± 2.9  12.8 ± 3.2  8.3 ± 2.9 |
| Protein (energy%) | 16.4 ± 4.1 | 16.4 ± 3.4 | 16.1 ± 2.9 |
| CHO^1^ (energy%) | 41.0 ± 5.7 | 42.3 ± 6.4 | 42.8 ± 6.1 |
| Alcohol (energy%) | 1.9 ± 2.7 | 1.8 ± 2.3 | 1.7 ± 2.4 |
| Fibre (g/day) | 23.4 ± 10.0 | 24.1 ± 5.6 | 24.2 ± 6.0 |
| Cholesterol (mg/day) | 204 ± 66 | 201 ± 56 | 196 ± 64 |
| Vitamin E (mg/day)^2^ | 13.9 ± 7.4 | 13.2 ± 5.3 | 13.1 ± 5.5 |

Table 1. Composition of the daily background diet during the three study periods excluding the provided supplements. Values are means ± SD. All subjects (n=20) received the three dietary periods in random order. ^1^SAFA: saturated fatty acids, MUFA: monounsaturated fatty acids, PUFA: polyunsaturated fatty acids, CHO: carbohydrates. ^2^Dietary vitamin E intake without capsule supplementation.

|  | Control period | Vitamin E period | Lipoic acid period |
| --- | --- | --- | --- |
| Vitamin E (μg/mL) | 16.5 ± 4.0 | 37.4 ± 10.4^1^ | 17.5 ± 4.6 |

Table 2. Plasma vitamin E concentrations at the end of all three intervention periods. Values are means ± SD (n=20). Significantly different compared with control period and lipoic acid period ^1^(P<0.001).

|  | Control period | Vitamin E period | Lipoic acid period |
| --- | --- | --- | --- |
| Inflammation | | | |
| CRP (g/L) | 2.75 ± 2.49 | 2.33 ± 1.60 | 3.58 ± 4.78 |
| IL-6 (ng/mL) | 1.18 ± 0.45 | 1.21 ± 0.52 | 1.18 ± 0.47 |
| TNFα (ng/mL) | 3.65 ± 1.04 | 3.67 ± 1.02 | 3.79 ± 0.95 |
| Thrombomodulin (ng/mL) | 5.11 ± 1.38 | 5.04 ± 1.40 | 5.05 ± 1.34 |
| SAA (mg/L) | 8.88 ± 11.40 | 6.05 ± 4.56 | 8.47 ± 5.30 |
| Cellular adhesion | | | |
| E-selectin (ng/mL) | 27.00 ± 9.67 | 30.01 ± 12.93 | 29.74 ± 13.17 |
| P-selectin (ng/mL) | 65.54 ± 18.65 | 68.30 ± 22.18 | 69.79 ± 24.74 |
| sICAM-1 (ng/mL) | 267.75 ± 55.85 | 269.55 ± 60.37 | 277.40 ± 65.36 |
| sICAM-3 (ng/mL) | 0.58 ± 0.15 | 0.62 ± 0.17 | 0.60 ± 0.17 |
| VCAM-1 (ng/mL) | 428.45 ± 117.41 | 426.00 ± 119.21 | 423.55 ± 98.91 |
| Iron and copper status | | | |
| Total iron (μmol/l) | 14.48 ± 4.32 | 15.72 ± 4.83 | 15.69 ± 5.83 |
| Ferritin (μg/L) | 140.95 ± 199.80 | 139.48 ± 166.61 | 147.48 ± 174.53 |
| Transferrin (g/L) | 2.74 ± 0.46 | 2.70 ± 0.37 | 2.68 ± 0.38 |
| Transferrin saturation (%) | 21.96 ± 8.02 | 24.08 ± 9.22 | 24.10 ± 10.22 |
| Copper (μmol/L) | 12.24 ± 1.64 | 11.93 ± 1.61 | 12.02 ± 1.51 |
| Ceruloplasmin (g/L) | 0.25 ± 0.05 | 0.24 ± 0.02 | 0.24 ± 0.03 |

Table 3. Effect of vitamin E and lipoic acid supplementation on plasma inflammatory markers, cellular adhesion molecules and iron and copper status parameters vs. control period. Values are means ± SD (n=20).

| Age (years) | 63.1 ± 5.8 |
| --- | --- |
| Male / Female (n) | 16 / 4 |
| IGT / DM2^1^ | 12 / 8 |
| BMI (kg/m2) | 29.5 ± 3.7 |
| Glucose (mmol/L) | 7.3 ± 1.6 |
| HbA1c (%) | 6.2 ± 0.9 |
| Total cholesterol (mmol/L) | 5.3 ± 0.9 |
| LDL cholesterol (mmol/L) | 3.3 ± 0.8 |
| HDL cholesterol (mmol/L) | 1.3 ± 0.3 |
| Triacylglycerol (mmol/L) | 1.5 ± 0.6 |

Table 4. Subject characteristics. Values are means ± SD (n=20). ^1^IGT: impaired glucose tolerance, DM2: type 2 diabetes.
