# Supplementary material for: The effects of vitamin E or lipoic acid supplementation on oxyphytosterols in subjects with elevated oxidative stress: a randomized trial
Source: Sci Rep. 2017 Nov 10;7:15288. doi: 10.1038/s41598-017-15615-y (PMC5681676; doi:10.1038/s41598-017-15615-y)
Supplement: Supplementary file 3 — PROTOCOL [file 41598_2017_15615_MOESM3_ESM.pdf]

The effects of consumption of vitamin E or lipoic acid on serum oxyphytosterol concentrations in type II diabetic patients

Written by:
Sabine Baumgartner
Ronald P. Mensink
Jogchum Plat
Department of Human Biology
Nutrition and Toxicology Research Institute Maastricht
Maastricht University
P.O. Box 616
6200 MD Maastricht
The Netherlands
Telephone: +31-43-3881309
Fax: +31-43-3670976
E-mail: j.plat@maastrichtuniversity.nl

PROTOCOL TITLE

The effects of consumption of vitamin E or lipoic acid on serum oxyphytosterol concentrations in type II diabetic patients

De effecten van de inname van vitamine E of liponzuur op de oxyphytosterolen concentraties in het bloed van patiënten met diabetes type II

**Protocol ID**	PRJIP11_TOP_METC
**Short title**	Antioxidants and oxyphytosterol concentrations
**Version**	1.0
**Date**	30-07-2012
**Coordinating investigator**	Sabine Baumgartner, MSc<br>Department of Human Biology<br>School for Nutrition, Toxicology and Metabolism<br>Maastricht University<br>P.O. Box 616<br>6200 MD Maastricht<br>The Netherlands<br><br>Telephone: +31-43-3881313<br>Fax: +31-43-3670976<br>E-mail: sabine.baumgartner@maastrichtuniversity.nl
**Principal investigator(s)**	Ronald P. Mensink, PhD<br>Telephone: +31-43-38813<br>E-mail: r.mensink@maastrichtuniversity.nl<br><br>Jogchum Plat, PhD<br>Telephone: +31-43-3881309<br>E-mail: j.plat@maastrichtuniversity.nl
**Project team members**	Aalt Bast, PhD<br>Guido R.M.M. Haenen, PhD<br>Nina A.W.M. Wystyrk, dietician<br>Maud M.H. Beckers, technician
**Sponsor (in Dutch: verrichter/opdrachtgever)**	Maastricht University Medical Center<br>NUTRIM School for Nutrition, Toxicology and Metabolism
**Independent physician(s)**	Tineke van de Weijer, MD<br>Department of Human Biology<br>Maastricht University<br>P.O. Box 616<br>6200 MD Maastricht<br>The Netherlands<br>Telephone: +31-43-3881311<br>E-mail: t.vandeweijer@maastrichtuniversity.nl

PROTOCOL SIGNATURE SHEET

Name	Signature	Date
**Head of Department:**<br>**Ronald Mensink, PhD**		
**Principal Investigator:**<br>**J.Plat, PhD**		

TABLE OF CONTENTS

1. INTRODUCTION AND RATIONALE 8
1.1 Oxyphytosterols 8
1.2 Oxidative stress and diabetes 9
1.3 Vitamin E and lipoic Acid 9
2. OBJECTIVES 10
3. STUDY DESIGN 10
4. STUDY POPULATION 12
4.1 Population 12
4.2 Screening visit 12
4.3 Inclusion criteria 12
4.4 Exclusion criteria 13
4.5 Sample size calculation 13
5. TREATMENT OF SUBJECTS 14
5.1 Investigational products 14
5.2 Product handling and storage 14
6. METHODS 15
6.1 Study parameters/endpoints 15
6.1.1 Metabolic risk markers 15
6.1.2 Food intake 16
6.1.3 Body weight 16
6.2 Randomisation, blinding and treatment allocation 16
6.3 Study procedures 17
6.4 Withdrawal of individual subjects 19
6.5 Replacement of individual subjects after withdrawal 19
6.6 Follow-up of subjects withdrawn from treatment 19
7. SAFETY REPORTING 19
7.1 Section 10 WMO event 19
7.2 Adverse and serious adverse events 19
7.3 Follow-up of adverse events 20
8. STATISTICAL ANALYSIS 20
8.1 Descriptive statistics 20
8.2 Univariate analysis 20
9. ETHICAL CONSIDERATIONS 21
9.1 Regulation statement 21
9.2 Recruitment and consent 21
9.3 Privacy 21
9.4 Benefits and risks assessment, group relatedness 21
9.5 Compensation for injury 22

9.6 Incentives (if applicable) 22

10. ADMINISTRATIVE ASPECTS AND PUBLICATION 23

10.1 Handling and storage of data and documents 23

10.2 Amendments 24

10.3 End of study report 24

10.4 Public disclosure and publication policy 24

11. REFERENCES 24

APPENDIX 1: Study design........26

APPENDIX 2: Format label text........27

APPENDIX 3: Certificates of analysis of the investigational products........28

APPENDIX 4: Product specification files........34

APPENDIX 5: QA contract/technical agreement........39

LIST OF ABBREVIATIONS AND RELEVANT DEFINITIONS

AE	Adverse Event
ApoA-I	Apolipoprotein A-I
ApoB100	Apolipoprotein B100
CCMO	Central Committee on Research Involving Human Subjects; in Dutch: Centrale Commissie Mensgebonden Onderzoek
CV	Curriculum Vitae
DMII	Diabetes Type 2
GCP	Good Clinical Practice
GMP	Good Manufacturing Practice
HDL-C	High-density lipoprotein cholesterol
hsCRP	High-sensitive C-reactive protein
IL-6	Interleukin-6
MCP-1	Monocyte chemotactic protein-1
METC	Medical research ethics committee (MREC); in Dutch: medisch ethische toetsing commissie (METC)
(S)AE	(Serious) Adverse Event
sE-Selectin	Soluble E-Selectin
sICAM-1	Soluble Intercellular adhesion molecule 1
Sponsor	The sponsor is the party that commissions the organisation or performance of the research, for example a pharmaceutical company, academic hospital, scientific organisation or investigator. A party that provides funding for a study but does not commission it is not regarded as the sponsor, but referred to as a subsidising party.
TAG	Triacylglycerol
TBARS	Thiobarbituric acid reactive substances
TCH	Total cholesterol
TNF-α	Tumor necrosis factor alpha
TNFR	Tumor necrosis factor receptor
VCAM-1	Vascular cell adhesion molecule 1
Wbp	Personal Data Protection Act (in Dutch: Wet Bescherming Persoonsgevens)
WMO	Medical Research Involving Human Subjects Act (in Dutch: Wet Medisch-wetenschappelijk Onderzoek met Mensen

SUMMARY

Rationale: Plant sterols can oxidize, which results in the formation of oxyphytosterols. Animal studies have now suggested that oxyphytosterols are atherogenic, but this relation has not yet been studied in humans. In our previous study (METC 09-3-088) we have shown in healthy volunteers that serum oxyphytosterol concentrations are linked to oxidative stress status (i.e. we were able to identify high and low sterol oxidizers). Type II diabetic patients are characterized by increased oxidative stress markers and reduced antioxidant capacity. Therefore, we want to evaluate the oxyphytosterol concentrations in this population. Moreover, we propose to evaluate the effect of antioxidant supplementation, i.e. vitamin E or lipoic acid, on serum oxyphytosterol concentrations in type II diabetic patients. Lowering oxyphytosterol concentrations in this population would obviously beneficial in case oxyphytosterols turn out to be atherogenic.

Objective: The objective of the present study is to examine the effect of consuming vitamin E (900 mg) or lipoic acid (600 mg) for 4 weeks on fasting oxyphytosterol concentrations in type II diabetic patients.

Study design: A randomized, double blind, placebo-controlled crossover design. The total study duration will be 20 weeks, consisting of 3 test periods of 4 weeks in which subjects will use the investigational products. Each period will be separated by a washout period of 4 weeks.

Study population: 20 diabetic male and female patients, aged 18-75 years.

Intervention: Subjects will be asked to consume three times daily control capsules, vitamin E capsules (total 900 mg) or lipoic acid capsules (total 600 mg) for three periods of four weeks. They will be asked to consume these capsules daily, divided over three eating moments. Each test-period is separated by a washout period of 4 weeks, during which the subjects will return to their habitual dietary patterns. They are not allowed to consume any dietary supplements during the entire study, including the washout periods. In total they will visit the department on 9 occasions to give a blood sample.

Main study parameters/endpoints: Blood samples will be drawn at weeks 1, 3 and 4 of each test period. The samples will be analysed for serum / plasma concentrations of plant sterols, oxyphytosterols, oxidative stress markers, antioxidant capacity, lipoproteins, and for markers reflecting low-grade systemic inflammation and endothelial dysfunction.

Nature and extent of the burden and risks associated with participation, benefit and group relatedness: Blood samples will be drawn on 9 different occasions in a time frame of 20 weeks with a total amount of 130.5 mL. During the screening procedure 11 mL (2x 5.5 ml) blood will be sampled. Furthermore, subjects will be asked to fill out a food frequency questionnaire three times at the end of each experimental period. Apart from a

haematoma or bruise, which can occur during or after venepuncture, no side effects of the intervention itself are expected.

1. INTRODUCTION AND RATIONALE

1.1 Oxyphytosterols

Oxyphytosterols are the oxidized form of plant sterols in the circulation. Plant sterols are normal components in our daily diet and they exert the same cellular functions in plants as cholesterol does in animals. The average intake of plant sterols in Western countries is approximately 250 mg/day, mainly derived from vegetable oils, cereals, nuts, seeds, fruits and vegetables. Plant stanols are the saturated derivatives of plant sterols. Plant sterols and stanols are structurally related to cholesterol, but they have a different side-chain configuration. As humans are unable to synthesize plant sterols and stanols, their serum concentrations are mainly determined by intestinal absorption and due to their low absorption; serum concentrations are less than 1% of that of serum cholesterol (1,2).
As mentioned above, plant sterols and cholesterol are structurally related, and therefore both can be oxidized and give rise to oxidation products. The side-chain of cholesterol can be oxidized enzymatically and the nucleus non-enzymatically, which is probably a radical driven process. For plant sterols, only the nucleus can be oxidized. Due to sterical hindrance caused by the different side-chain structure of plant sterols as compared to cholesterol, the side-chain of plant sterols cannot be oxidized. The ring-oxidation products are potentially atherogenic, at least for cholesterol oxidation products (3). The most important oxyphytosterols that have been identified are; 5α,6α-epoxy-sitosterol, 7=O-sitosterol, 7β-OH-sitosterol, en 3β,5α,6β-tri-hydroxy-sitosterol. The same oxyphytosterols can be identified for campesterol (4). Research concerning the location where oxyphytosterols are produced, on the breakdown route and speed or on the pathophysiological effect, is still lacking unfortunately.
The concentrations of oxyphytosterols in human plasma have so far been measured in two cross-sectional studies and in two intervention studies. In a first study by Plat et al. (4) oxyphytosterols could be identified in serum of sitosterolaemic patients, but not in serum from healthy volunteers. Later, Grandgirard et al. used a quantification method with a lower limit of detection and were able to identify concentrations of oxyphytosterols in plasma from healthy volunteers (5). In the mean time the methodology to analyse serum oxyphytosterol concentrations has much improved. We have recently determined serum oxyphytosterol concentrations in 45 healthy human subjects, who consumed 3.0 g/d plant sterols and plant stanols as part of their daily diet for 4 weeks (METC 09-3-088). Results of this study indicated that neither daily consumption of a plant sterol-enriched margarine

nor a plant stanol-enriched margarine changed oxyphytosterol concentrations when compared to control margarine. However, additional analyses in this study showed that oxyphytosterol concentrations were highly correlated with oxidized LDL concentrations and oxysterol concentrations, which are both sensitive markers for oxidative stress. Interestingly, we were able to identify consistent high and low sterol oxidizers. This is of course a highly relevant finding in the light of potential atherogenicity of oxyphytosterols.

1.2 Oxidative stress and diabetes

Oxidative stress is defined as an increased production of reactive oxygen species (ROS) and/or reduced antioxidant capacity and is thought to play a central role in the development of atherosclerosis and plaque formation (6,7). Reducing oxidative stress by means of antioxidants has major health benefits and is associated with a lower rate of coronary heart disease (8,9). Several chronic diseases such as diabetes mellitus type 2 (DMII) are characterized by an increased oxidative stress status (10).

DMII is one of the leading chronic diseases worldwide and a major risk factor for the development of cardiovascular diseases. Oxidative stress is associated with the development and progression of diabetes and its complications (11). Studies have shown that DMII patients have elevated serum oxysterol concentrations (cholesterol oxidation products) when compared to healthy subjects (12, 13). Due to the similarity in structure between oxysterols and oxyphytosterols and the correlation between serum oxysterols and oxyphytosterols, we would expect that DMII patients would also have elevated serum oxyphytosterol concentrations. This has however never been evaluated, but is relevant knowledge since in vitro studies and animal studies have shown that oxyphytosterols themselves might exert atherogenic properties, highlighting the importance of reducing serum oxyphytosterol concentrations (14,15).

1.3 Vitamin E and lipoic Acid

As mentioned above, consumption of antioxidant supplements is able to reduce oxidative stress and might eventually lower CVD. Vitamin E is one of the most well known dietary antioxidants and studies have shown that consuming vitamin E, results in reduced oxidative stress markers (16,17). While previous research has shown that serum oxysterol concentrations can be reduced via vitamin E consumption (18), the effect on serum oxyphytosterol concentrations has never been evaluated. Besides vitamin E other antioxidants also received a lot of attention. Evidence is emerging concerning the beneficial effects of lipoic acid. Lipoic acid is a short chain fatty acid that is present in nature and produced endogenously in mammals, where it has a role as a cofactor for

mitochondrial α-ketoacid dehydrogenases. It has strong antioxidant capacities and has been shown to reduce oxidative stress in humans (19). In addition, lipoic acid is able to increase insulin sensitivity in DMII patients and to alleviate diabetic neuropathy (20,21) and is available as dietary supplement. Vitamin E is a fat-soluble antioxidant while lipoic acid is both water-soluble and fat-soluble. To increase the knowledge of oxyphytosterols as an oxidative stress marker, it is relevant to compare two antioxidants that differ regarding their solubility, i.e. vitamin E and lipoic acid.

In our previous study, we have shown that serum oxyphytosterol concentrations are strongly linked to oxidative stress status. By evaluating the effect of antioxidant supplementation on serum oxyphytosterol concentrations, the role of oxyphytosterols as oxidative stress makers will be confirmed. We want to evaluate this effect in DMII patients, as this population is characterized by increased oxidative stress markers and reduced antioxidant capacity.

2. OBJECTIVES

The objective of the present study is to examine the effect of consuming vitamin E (900 mg/day) or lipoic acid (600 mg/day) for 4 weeks on fasting oxyphytosterol concentrations in DMII patients.

The null hypothesis (H_0) is:
In DMII patients, consuming vitamin E (900 mg) or lipoic acid (600 mg) for 4 weeks does not change oxyphytosterol concentration as compared to a control condition.

The alternative hypothesis (H_a) is:
In DMII patients, consuming vitamin E (900 mg) or lipoic acid (600 mg) for 4 weeks changes oxyphytosterol concentration as compared to a control condition.

3. STUDY DESIGN

This study is a randomized, placebo-controlled, crossover study with 20 type II diabetic patients (both men and women). Subjects will be recruited in and near the vicinity of Maastricht by means of posters distributed in university and hospital buildings, advertisements in local newspapers, the hospital bulletin and on the internet (www.digi-prik.nl). Subjects who have participated in earlier studies and indicated to be interested in participation in other studies will be contacted. Before screening, subjects will be informed about the procedures during the study via oral and written information (see information brochure). After information is given, subjects can consider participation for at least 3

days. When positive, informed consent will be obtained before start of the study. Participation will be on voluntary basis and people who are willing to participate will be invited for a screening visit. Subjects will be informed about their results obtained during the screening and advised to consult their general practitioner when values of the screening parameters are outside the normal ranges. Subjects have to state specifically on the informed consent page that they do not object to receiving data on accidental findings. When subjects fulfil all inclusion criteria, they may enter the study.

The intervention will consist of control capsules, vitamin E capsules with a daily intake of 900 mg, and lipoic acid capsules with a daily intake of 600 mg. These doses are based upon previous research showing reduced oxidative stress at these intakes (19,22). During the first 4 weeks of the study (period I), subjects will be provided with the control capsules, the vitamin E capsules or the lipoic acid capsules. After period I, the subjects will return to their normal pattern (indicating no vitamin supplementation) for 4 weeks (washout period). Then, the subjects will cross over to another dietary regime in period II. Hereafter they will again return to their normal eating habits for 4 weeks and cross over to the last dietary regime in period III. There will be no dietary restriction as long as dietary habits remain stable over the entire study. During the entire study period (including the washout periods), the subjects are not allowed to consume any dietary supplements other than those provided by us. All subjects must adhere to each dietary regime, meaning consumption of control capsules, vitamin E capsules and lipoic acid capsules during the three periods. Compliance can be monitored by measurement of serum vitamin E and lipoic acid concentrations.

As depicted in table 1 (Appendix 1), the subjects will come to the department at the beginning of period I, II and III (weeks 1, 9, and 17), after three weeks of each period (weeks 3, 11 and 19) and at the end of each period (weeks 4, 12 and 20). During these visits, a fasting blood sample will be taken and body weight is measured. Based on earlier studies we expect a washout period of 4 weeks to be long enough. All subjects will pick up their supply of products when they visit the department for the blood sampling at the beginning of period I, II and III. The capsules will be provided in bottles containing one-week portions of 23 capsules (3 capsules a day, and 2 extra capsules). Capsules that are left over at the end of the week must be returned to the department and will be counted. Capsules are similarly packed to assure blinding of the subjects and investigators. Moreover, the bottles will be color-coded to blind both the subjects and the investigators. The subjects will record in a diary the amounts and times of consumption of the capsules and they will also be asked to record every signs of illnesses, medication used and any deviations from protocol. Furthermore, they will be urged not to change their dietary habits, level of physical activity, use of alcohol or of oral contraceptives throughout the

study. A registered dietician will check these dairies at each visit. At the end of the three periods I, II and III, the subjects will also fill out a food frequency questionnaire to estimate their habitual intake over the previous 4 week period (weeks 4, 12, 20).

4. STUDY POPULATION

4.1 Population

Subjects will include 20 type II diabetic patients, aged between 18 and 75 years that have to fulfil the inclusion criteria as described below. From our previous experiences, we know that a high proportion of people of >75 years do have one or more of the exclusion criteria as formulated in paragraph 4.4. Because we do not want to encumber these subjects unnecessarily, we have decided to set the upper limit for age at 75 years.

4.2 Screening visit

The subjects will be invited for a screening visit, this visit will include recording of:

- body weight
- length
- blood pressure (diastolic and systolic)
- blood parameters (serum total cholesterol, triacylglycerol and glucose concentrations)
- use of medication
- current disease and history of coronary heart disease

Subjects will come to the department twice for two screening visits. During the first screening visit, the subjects will be weighed, blood pressure will determined in four-fold (the first measurement will be discarded and the last three measurement will be averaged), body height will determined and a venous blood sample (1*3.5 ml, 1*2 ml) will be drawn for analysis of serum total cholesterol, triacylglycerol, glucose concentrations and liver function markers.

During the second screening visit, body weight and blood pressure will be determined again and a second venous blood sample (1*3.5 ml and 1*2 ml) will be drawn for analysis of serum total cholesterol, triacylglycerol and glucose concentrations.

4.3 Inclusion criteria

The inclusion criteria are:

- aged between 18 and 75 years
- Body Mass Index (BMI) between 20-30 kg/m^2
- mean serum total cholesterol < 8.0 mmol/L

- mean serum triacylglycerol < 3.0 mmol/L
- diagnosed with diabetes mellitus type 2 on a clinical basis

4.4 Exclusion criteria

The exclusion criteria are:

- unstable body weight (weight gain or loss > 3 kg in the past two months)
- active cardiovascular diseases like congestive heart failure or recent (<6 months) event (acute myocardial infarction, cerebral vascular incident)
- severe medical conditions that might interfere with the study such as epilepsy, asthma, chronic obstructive pulmonary disease, inflammatory bowel disease and rheumatoid arthritis
- use of medication such as corticosteroids, diuretics or lipid lowering therapy
- use of insulin therapy
- abuse of drugs or alcohol (>21 units per week)
- not willing to stop the consumption of dietary supplements 1 month before the start of the study (wash-in period)
- use of an investigational product within another biomedical study within the previous month
- pregnant or breast-feeding women
- current smoker

4.5 Sample size calculation

In our previous study, we were able to detect a decrease of 0.07 ng/mL in 7b-OH-campesterol concentrations in healthy volunteers upon plant stanol consumption. A larger decrease in oxyphytosterol concentrations is expected upon vitamin E and lipoic acid consumption, as they are antioxidants and research has already shown that they are able to decrease oxidative stress markers. A recent study found a decrease of 28% in oxycholesterol concentrations upon vitamin E consumption [18]. We would expect larger decreases upon vitamin E and lipoic acid consumption in DMII patients, as they are characterized by an increased oxidative stress status and changes will be larger if baseline concentrations are higher. Therefore, we would expect a change of 0.15 ng/mL in plasma 7b-OH-campesterol concentrations in DMII patients. Using this true change of 0.15 ng/mL and the known within-subject variation on the response of 0.14 ng/mL, it can be calculated that we need 18 subjects to have a power of 80% to detect the indicated difference, if we consider a P-value < 0.017 (to account for multiple comparisons between

groups) to be statistically significant. The expected dropout rate is 10%. Therefore, 20 diabetic patients will be recruited.

5. TREATMENT OF SUBJECTS

Subjects will be asked to consume three times daily (one at breakfast one at lunch and one at dinner) either control capsules, vitamin E capsules (900 mg) or lipoic acid capsules (600 mg) for three periods of four weeks. Each test period is separated by a washout period of 4 weeks, during which the subjects will return to their normal pattern (indicating no vitamin supplementation). During the entire study period (including the washout periods), the subjects are not allowed to consume any dietary supplements other than those provided by us. They will visit the department on 9 occasions (weeks 1, 3, 4, 9, 11, 12, 17, 19 and 20) to donate a blood sample, which is explained into more detail in §6.3.

5.1 Investigational products

Basic Pharma (Geleen, the Netherlands) will produce vitamin E, lipoic acid and matching placebo capsules to ensure the double-blind design. Production will be according to GMP and appropriate testing to ensure quality will be performed. The certificates of analysis are found in Appendix 3, the product specification files are found in Appendix 4 and the technical and quality assurance agreements with Basic Pharma are found in Appendix 5.

Vitamin E: Vitamine E capules will be administered orally, 1 capsule of 300mg, 3 times per day, equivalent to 900mg daily. Subjects will be advised to take the capsule after each meal with a glass of water.

Lipoic acid: Lipoic acid capules will be administered orally, 1 capsule of 200mg, 3 times per day, equivalent to 600mg daily. Subjects will be advised to take the capsule after each meal with a glass of water.

Placebo: Placebo capsules will be filled with gelatine, cellulosum microcristallinum PH102 and will also be administered orally 3 times per day using the same instructions as described for vitamin E and lipoic acid.

5.2 Product handling and storage

Vitamin E, lipoic acid and placebo capsules will be packed into separate boxes labelled with the number of capsules to be taken daily for a period of 1 week, the format for these labels is presented in appendix 2. One box will contain 23 capsules (which is 2 more than necessary) and labelled as box A, B or C. Labelling A, B and C is used to blind the capsules for investigator and subjects. Labelled boxes will be shipped to the Department of Human Biology, MUMC+. The boxes will be stored in the storage room (room 0.325a)

of the metabolic kitchen of the Metabolic Research Unit Maastricht (MRUM). They will be stored in a dry location, below 25 °C, protected from exposure to environmental changes. At the end of the study the boxes must be returned to the researchers to calculate (non)-compliance.

6. METHODS

6.1 Study parameters/endpoints

We will analyse parameters related to the research questions as described below. Based on new insights related to the original research question, it may be decided to analyse the samples for other parameters. Therefore, subjects will explicitly be asked in the informed consent form to consent (or not) with this approach.

6.1.1 Metabolic risk markers

- Oxidative stress markers

Butylated hydroxy toluene (BHT) will be added to the EDTA blood collection tubes to prevent auto-oxidation of the sterols and other components in the blood.
The following oxidative stress markers will be determined in plasma:

- Plasma oxyphytosterol concentrations, measured in week 1, 4, 9, 12, 17 and 20
- Plasma oxysterol concentrations, measured in week 1, 4, 9, 12, 17 and 20
- Plasma oxidized LDL concentrations, measured in week 1, 4, 9, 12, 17 and 20
- TBARS assay, measured in week 1, 4, 9, 12, 17 and 20

- Antioxidant capacity

The following antioxidant concentration will be measured to determine compliance of the subjects and to determine antioxidant capacity:

- Plasma vitamin E concentrations, measured in week 1, 4, 9, 12, 17 and 20
- Plasma lipoic acid concentrations, measured in week 1, 4, 9, 12, 17 and 20
- Glutathion concentrations, measured in week 4, 12 and 20
- Plasma uric acid concentrations, measured in week 4, 12 and 20
- Trolox antioxidant capacity, measured in week 4, 12 and 20

- Lipid and lipoprotein concentrations

The following lipid and lipoprotein concentrations will be measured to assess determinants of the lipid metabolism:

- TCH concentrations, measured in week 1, 3, 4, 9, 11, 12, 17, 19 and 20
- HDL-C concentrations, measured in week 1, 3, 4, 9, 11, 12, 17, 19 and 20

- TAG concentrations, measured in week 1, 3, 4, 9, 11, 12, 17, 19 and 20
- ApoB100 concentrations, measured in week 1, 3, 4, 9, 11, 12, 17, 19 and 20
- ApoA-1 concentrations, measured in week 1, 3, 4, 9, 11, 12, 17, 19 and 20

• Glucose metabolism
  - Glucose concentrations, measured in week 4, 12 and 20

• Low-grade inflammation and endothelial activity

The following parameters will be measured to assess concentrations of low-grade inflammation and endothelial activity:

- hsCRP, TNF-α, TNF-RI, TNFR-II, IL-6, MCP-1, sICAM-1, sVCAM-1 and sE-selectin, measured in week 4, 12 and 20

• Iron and copper status

The following markers will be measured to assess iron and copper status. Iron and copper are the main metals present in human sera, and an increased metal status could indicate a higher oxidative susceptibility.

- Total iron, measured in week 4, 12 and 20
- Transferrine, measured in week 4, 12 and 20
- Ferritine, measured in week 4, 12 and 20
- Copper, measured in week 4, 12 and 20
- Ceruloplasmin, measured in week 4, 12 and 20

6.1.2 Food intake

Habitual food intake will be measured at the end of each test period using a validated food frequency questionnaire, which will be checked in the presence of a dietician immediately.

6.1.3 Body weight

Body weight without shoes and heavy clothing will be measured during each visit to the department.

6.2 Randomisation, blinding and treatment allocation

The subjects will receive the three experimental periods in a randomized order, based upon a computer-generated table with random numbers. For this, a categorical list in logical order will be created including all interventions per subject. After addition of a

computer-generated list of random numbers, the list will be sorted by subject number and random number, resulting in a randomized list of treatment allocation. The randomization code will be broken after statistical analyses are completed. For randomization, subjects will be stratified for gender. To blind the researchers and the subjects, the capsules will be packed and coded by Basic Pharma, an independent company.

6.3 Study procedures

Subjects will be asked to consume three times daily control capsules, or vitamin E capsules or lipoic acid capsules for three periods of four weeks. They will be asked to consume these capsules daily, divided over the three eating moments (breakfast, lunch an dinner). Each test-period is separated by a wash-out period of 4 weeks, during which the subjects will return to their normal eating pattern (indicating no vitamin supplementation), in which they are not allowed to consume vitamin E or lipoic acid supplements.

They will visit the department at weeks 1,3, 4, 9, 11, 12, 17, 19 and 20 to give a blood sample. At weeks 1, 9 and 17 they will also receive a new supply of capsules and at weeks 4, 12 and 20 they will fill out a food frequency questionnaire. Their body weight is measured during each visit to the department.

The amount of blood sampled at each occasion is given in table 2.

Table 2. Blood sampling scheme

**Week**	**1**	**3**	**4**	**9**	**11**	**12**	**17**	**19**	**20**
**Action**	1 blood sample	1 blood sample	1 blood sample	1 blood sample	1 blood sample	1 blood sample	1 blood sample	1 blood sample	1 blood sample
**mL**	13.5	3.5	26.5	13.5	3.5	26.5	13.5	3.5	26.5

The total amount of blood drawn will be 130.5 ml per person during the whole study, divided over a period of 20 weeks with 9 sampling moments.

Blood samples drawn in week 1, 9 and 17 will be used to check whether the wash-out period has been successful. Samples drawn in week 3 and 4, 11 and 12, and 19 and 20 are needed to obtain a reliable determination of serum lipid concentrations, as these tend to fluctuate over time. Table 3 list the amounts of blood needed to perform the chosen analysis for this study.

Table 3. Blood analysis protocol for single blood sampling

**Week 1, 9, 17**	
Serum	3.5 mL
Lipids	250 µL
ApoB100/ApoA1	250 µL
2*spare	2*250 µL
EDTA + BHT	8.0 mL
Oxyphytosterols	1000 µL
Oxysterols	1000 µL
2*spare	2*1000 µL
EDTA	2.0 mL
Vitamin E	250 µL
Lipoic acid	250 µL
2*spare	2*250 µL
**Week 3, 11, 19**	
Serum	3.5 mL
Lipids	250 µL
ApoB100/ApoA1	250 µL
2*spare	2*250 µL
**Week 4, 12, 20**	
Serum	8.5 mL
Lipids	250 µL
ApoB100/ApoA1	250 µL
Inflammation	500 µL
Endothelial activity	500 µL
Iron and copper status	500 µL
3*spare	3*250 µL
EDTA + BHT	10.0 mL
Oxyphytosterols	1000 µL
Oxysterols	1000 µL
Oxidized LDL	250 µL
TBARS assay	250 µL
F2-isoprostanes	250 µL
2*spare	2*250 µL
spare	1000 µL
EDTA	6.0 mL
Vitamin E	250 µL
Lipoic acid	250 µL
Uric acid	250 µL
TEAC	250 µL
2*spare	3*250 µL

EDTA whole blood	2.0 mL
Glutathion	250 µL
Vitamin C	250 µL
2*spare	2*250 µL
NaF	2.0 mL
Glucose	250 µL
2*spare	2*250 µL

6.4 Withdrawal of individual subjects

Subjects can leave the study at any time for any reason if they wish to do so without any consequences. The investigator can decide to withdraw a subject from the study for urgent medical reasons.

6.5 Replacement of individual subjects after withdrawal

After withdrawal, subjects will not be replaced.

6.6 Follow-up of subjects withdrawn from treatment

After withdrawal, no follow-up of subjects will take place. In case of withdrawal due to medical compliance, subjects will be referred to a general practitioner.

7. SAFETY REPORTING

7.1 Section 10 WMO event

In accordance to section 10, subsection 1, of the WMO, the investigator will inform the subjects and the reviewing accredited METC if anything occurs, on the basis of which it appears that the disadvantages of participation may be significantly greater than was foreseen in the research proposal. The study will be suspended pending further review by the accredited METC, except insofar as suspension would jeopardise the subjects' health. The investigator will take care that all subjects are kept informed.

7.2 Adverse and serious adverse events

Adverse events are defined as any undesirable experience occurring to a subject during the study, whether or not considered related to the investigational product. All adverse events reported spontaneously by the subject or observed by the investigator or his staff will be recorded.

A serious adverse event is any untoward medical occurrence or effect that at any dose:
- results in death;
- is life threatening (at the time of the event);
- requires hospitalisation or prolongation of existing inpatients' hospitalisation;
- results in persistent or significant disability or incapacity;
- is a congenital anomaly or birth defect;
- is a new event of the trial likely to affect the safety of the subjects, such as an unexpected outcome of an adverse reaction, lack of efficacy of an IMP used for the treatment of a life threatening disease, major safety finding from a newly completed animal study, etc.

All SAEs will be reported through the web portal *ToetsingOnline* to the accredited METC that approved the protocol, within 15 days after the sponsor has first knowledge of the serious adverse reactions. SAEs that result in death or are life threatening should be reported expedited. The expedited reporting will occur not later than 7 days after the responsible investigator has first knowledge of the adverse reaction. This is for a preliminary report with another 8 days for completion of the report.

7.3 Follow-up of adverse events

All adverse events will be followed until they have abated, or until a stable situation has been reached. Depending on the event, follow up may require additional tests or medical procedures as indicated, and/or referral to the general physician or a medical specialist.

8. STATISTICAL ANALYSIS

8.1 Descriptive statistics

Data will be presented as mean values and standard deviation or mean change from baseline with 95% confidence interval.

8.2 Univariate analysis

Differences between the two experimental periods and the control period will be calculated and effects of the intervention will be examined by analysis of variance (ANOVA) test. A p-value <0.017 is considered to be statistically significant. All statistical analyses will be performed using SPSS 18.0 for Mac Os X (SPSS Inc., Chicago, IL, USA).

9. ETHICAL CONSIDERATIONS

9.1 Regulation statement

The study has to be approved by the Medical Ethical Committee of the University of Maastricht. The study will be conducted according to the principles of the Declaration of Helsinki (October 2008) and in accordance with the Medical Research Involving Human Subjects Act (WMO).

9.2 Recruitment and consent

Subjects will be recruited among men and women in and near the vicinity of Maastricht by means of posters distributed in university and hospital buildings, advertisements in local newspapers, the hospital bulletin and on the internet (www.digi-prik.nl). In addition, subjects who have participated in earlier studies and who have indicated that they are interested in other studies, will be sent an information brochure. Before the start of the study volunteers will be given oral and written information about the aim of the study. After information is given, subjects can consider participation for at least 3 days. Hereafter, informed consent will be obtained before start of the study. Participation will be on voluntary basis and people who are willing to participate will be invited for a screening visit. Subjects are free to approach the independent physician for further information and questions, and to withdraw at any stage of the study without further explanation.

9.3 Privacy

Before screening, subjects are informed about the procedures and informed consent will be obtained. Personal data will be handled confidentially and be stored in a password-protected file, to which only the investigators have access. The data will be stored for maximum 15 years. Samples will be coded and destroyed after 5 years. Only the investigators have access to the code. Subjects can leave the study at any time for any reason if they wish to do so without any consequences.

9.4 Benefits and risks assessment, group relatedness

The subjects will record in diaries any signs of illness, medication used, any deviations from the protocol, and any experienced adverse events (headache, stomach complaints, nausea, bloated feeling, flatulence, diarrhoea, constipation, itching, eruptions/rashes, fatigue, and dizziness).

Venepunctures can occasionally cause a local haematoma or a bruise. Some participants may report pain during venepuncture. There is no direct benefit for the participants.

9.5 Compensation for injury

The sponsor/investigator has a liability insurance that is in accordance with article 7, subsection 6 of the WMO.

The sponsor (also) has an insurance that is in accordance with the legal requirements in the Netherlands (Article 7 WMO and the Measure regarding Compulsory Insurance for Clinical Research in Humans of 23th June 2003). This insurance provides cover for damage to research subjects through injury or death caused by the study.

1. € 450.000,-- (i.e. four hundred and fifty thousand Euro) for death or injury for each subject who participates in the Research;
2. € 3.500.000,-- (i.e. three million five hundred thousand Euro) for death or injury for all subjects who participate in the Research;
3. € 5.000.000,-- (i.e. five million Euro) for the total damage incurred by the organisation for all damage disclosed by scientific research for the Sponsor as ‘verrichter’ in the meaning of said Act in each year of insurance coverage.

The insurance applies to the damage that becomes apparent during the study or within 4 years after the end of the study.

9.6 Incentives (if applicable)

The time invested by subjects is approximately 6 hours and 40 minutes, as can be seen in table 4. Compensation for participation will be €125,00. This amount is based on the fact that subjects have to come 9 times to the department for blood sampling and picking up capsules supplies and on the fact that they have to consume 3 capsules per day for 3 periods of 4 weeks (9 * 12,50 = 112,50), which can be rounded off upwards to €125,00. After withdrawal, the compensation will be pro rata. There will be no financial reward for the screening visits, as the time invested is minimal, while subjects will receive a free breakfast and a free health check. Traveling costs will be compensated (public transport or €0.19/km for car travel). The test products will be provided for free.

Table 4. Time investment of participants

Week	Blood sampling	Picking up supply and talk with dietician	Food frequency questionnaire	Total
-1	20 min			20 min
-2	20 min			20 min
1	20 min	10 min		30 min
3	20 min			20 min
4	20 min		30 min	50 min
9	20 min	10 min		30 min
11	20 min			20 min
12	20 min		30 min	50 min
17	20 min	10 min		30 min
19	20 min			20 min
20	20 min		30 min	50 min
Total time invested				6 hours and 40 min

10. ADMINISTRATIVE ASPECTS AND PUBLICATION

10.1 Handling and storage of data and documents

At the start of the study, subjects will be assigned a random number that will not change during the study. This number is linked with the name, address, date of birth, and telephone number of the subject in a password-protected file. Except for the technicians and the dietician, only members of the project team can access this file. For all other purposes, the random number will be used for subject identification. The data will be stored for maximum 15 years.

10.2 Amendments

Amendments are changes made to the research after a favourable opinion by the accredited METC has been given. All amendments will be notified to the METC that gave a favourable opinion.

10.3 End of study report

The investigator will notify the accredited METC of the end of the study within a period of 90 days. The end of the study is defined as the last patient's last visit.
In case the study is ended prematurely, the investigator will notify the accredited METC, including the reasons for the premature termination.

10.4 Public disclosure and publication policy

Publication policy is in agreement with the CCMO publication statement. The results of the study will be published in peer-reviewed scientific journals. Both positive and negative results of the study will be disclosed. The principal investigator will always try to publish and/or present results to the general public.

REFERENCES

1. Brufau G, Canela MA, Rafecas M. Phytosterols: physiologic and metabolic aspects related to cholesterol-lowering properties. Nutr Res. 2008 Apr;28(4):217-25.
2. Gylling H, Miettinen TA. The effect of plant stanol- and sterol-enriched foods on lipid metabolism, serum lipids and coronary heart disease. Ann Clin Biochem. 2005 Jul;42(Pt 4):254-63
3. Hovenkamp E, Demonty I, Plat J, Lutjohann D, Mensink RP, Trautwein EA. Biological effects of oxidized phytosterols: a review of the current knowledge. Prog Lipid Res. 2008 Jan;47(1):37-49.
4. Plat J, Brzezinka H, Lutjohann D, Mensink RP, von Bergmann K. Oxidized plant sterols in human serum and lipid infusions as measured by combined gas-liquid chromatography-mass spectrometry. J Lipid Res. 2001 Dec;42(12):2030-8.
5. Grandgirard A, Martine L, Demaison L, Cordelet C, Joffre C, Berdeaux O, et al. Oxyphytosterols are present in plasma of healthy human subjects. Br J Nutr. 2004 Jan;91(1):101-6.
6. Singh U, Jialal I. Oxidative stress and atherosclerosis. Pathophysiology. 2006 Aug;13(3):129-42
7. Münzel T, Gori T, Bruno RM, Taddei S. Is oxidative stress a therapeutic target in cardiovascular disease? Eur Heart J. 2010 Nov;31(22):2741-8.

8. Stampfer MJ, Hennekens CH, Manson JE, Colditz GA, Rosner B, Willet WC. Vitamin E consumption and the risk of coronary disease in women. N Engl J Med. 1993;328:1444-9

9. Rimm EB, Stampfer MJ, Ascherio A, Giovanucci EL, Colditz GA, Willet WC. Vitamin E consumption and the risk of coronary heart disease in men. N Engl J Med. 1993;328:1450-6

10. Maritim AC, Sanders RA, Watkins JB. Diabetes, oxidative stress, and antioxidants: a review. J Biochem Mol Toxicol. 2003;17:24-38

11. Ramkrishna V, Jailkhani R. Evaluation of oxidative stress in insulin dependent diabetes mellitus (IDDM) patients. Diagn Pathol. 2007;(2):22.

12. Federbar S, Pereira EC, Apolinario E, Bertolami MC, Faludi A, Monte O, et al. Cholesterol oxides as biomarkers of oxidative stress in type 1 and type 2 diabetes mellitus. Diabetes Metab Res Rev. 2007;(23):35-42.

13. Abo K, Mio T, Sumino K. Comparative analysis of plasma and erythrocyte 7-ketocholesterol as a marker for oxidative stress in patients with diabetes mellitus. Clin Biochem. 2000;33(7):541-547.

14. Adcox C, Boyd L, Oehrl L, Allen J, Fenner G. Comparative effects of phytosterol oxides and cholesterol oxides in cultured macrophage-derived cell lines. J Agric Food Chem. 2001 Apr;49(4):2090-5.

15. Maguire L, Konoplyannikov M, Ford A, Maguire AR, O'Brien NM. Comparison of the cytotoxic effects of beta-sitosterol oxides and a cholesterol oxide, 7beta-hydroxycholesterol, in cultured mammalian cells. Br J Nutr. 2003 Oct;90(4):767-75.

16. Gökkusu C, Palanduz S, Ademoglu E, Tamer S. Oxidant and antioxidant systems in NIDDM patients: influence of vitamin E supplementation. Endocr Res. 2001;27(3):377-86.

17. Hsu RM, Deveraj S, Jialal I. Autoantibodies to oxidized low-density lipoprotein in patients with type 2 diabetes mellitus. Clin Chem Acta. 2002;317(1-2):145-50.

18. Micheletta F, Natoli S, Miruraca M, Sbarigia E, Diczfalusy U, Iuliano L. Vitamin E supplementation in patients with carotid atherosclerosis: reversal of altered oxidative stress status in plasma but not in plaque. Arterioscler Thromb Vasc Biol. 2004 Jan;24(1):136-140

19. Shay KP, Moreau RF, Smith EJ, Smith AR, Hagen TM. Alpha-lipoic acid as a dietary supplement: Molecular mechanisms and therapeutic potential. Biochim Biophys Acta. 2009 Oct;1790(10):1149-60.

20. Zhang Y, Han P, Wu N, He B, Lu Y, Li S et al. Amelioration of lipid abnormalities by α-lipoic acid through antioxidative and anti-inflammatory effects. Obesity (Silver spring). 2011 Aug:19(8):1647-53.

21. McIlduff CE, Rutkove SB. Critical appraisal of the use of alpha lipoic acid (thioctic acid) in the treatment of symptomatic diabetic polyneuropathy. Ther clin Risk Manag. 2011;7:377-85.

22. Jackson Roberts II L, Oates JA, Linton MF, Fazio S, Meador BP, Gross MD, Shyr Y, Morrow JD. The relationship between dose of vitamin E and suppression of oxidative stress in humans. Free Radic Biol Med. 2007;43:1388-1393.

APPENDIX 1: Study design

Table 1. Study design

	**Period I**				Wash-out period	**Period II**				Wash-out period	**Period III**			
**Order I**	Control capsules					Vitamin E capsules					Lipoic acid capsules			
**Order II**	Control capsules					Lipoic acid capsules					Vitamin E capsules			
**Order III**	Vitamin E capsules					Lipoic acid capsules					Control capsules			
**Order IV**	Vitamin E capsules					Control capsules					Lipoic acid capsules			
**Order V**	Lipoic acid capsules					Control capsules					Vitamin E capsules			
**Order VI**	Lipoic acid capsules					Vitamin E capsules					Control capsules			
Week	1	2	3	4	5-8	9	10	11	12	13-16	17	18	19	20
Blood sampling	x		x	x		X		x	x		x		x	x
Food intake				x					x					x
Body weight	x		x	x		X		x	x		x		x	x
Picking up supply	x					x					x		x	

APPENDIX 2: Format label text

Protocol nr. PRJIP11_TOP_METC
Subject nr.: …..

23 capsules met vitamin E 300 mg, liponzuur 200 mg of placebo.
Voor oraal gebruik
3x daags, 1 capsules innemen met water (bij elke maaltijd)

Uitsluitend te gebruiken voor klinisch onderzoek.
Bewaren bij 15-25°C.
Buiten bereik en zicht van kinderen bewaren.
Verpakkingen meebrengen in week 4, 12 en 20 van het onderzoek.

Onderzoeker: Sabine Baumgartner
Universiteit Maastricht, Vakgroep Humane Biologie
Postbus 616, 6200 MD, Maastricht. T+31 433881313

Lotnr: ……. Her-testdatum: ……

APPENDIX 3: Certificates of analysis of the investigational products

APPENDIX 4: Product specification files

APPENDIX 5: QA contract/technical agreement
